# Tobacco Control and Health Advocacy in the European Union: Understanding Effective Coalition-Building

**DOI:** 10.1093/ntr/ntv016

**Published:** 2015-01-29

**Authors:** Heide Weishaar, Jeff Collin, Amanda Amos

**Affiliations:** 1 ^1^ MRC/CSO Social and Public Health Sciences Unit, University of Glasgow, Glasgow, UK;; 2 ^2^ Global Public Health Unit, School of Social and Political Science, University of Edinburgh, Edinburgh, UK;; 3 ^3^ Centre for Population Health Sciences, University of Edinburgh, Edinburgh, UK

## Abstract

**Introduction:**

Coalitions of supporters of comprehensive tobacco control policy have been crucial in achieving policy success nationally and internationally, but the dynamics of such alliances are not well understood.

**Methods:**

Qualitative semi-structured, narrative interviews with 35 stakeholders involved in developing the European Council Recommendation on smoke-free environments. These were thematically analyzed to examine the dynamics of coalition-building, collaboration and leadership in the alliance of organizations which successfully called for the development of comprehensive European Union (EU) smoke-free policy.

**Results:**

An alliance of tobacco control and public health advocacy organizations, scientific institutions, professional bodies, pharmaceutical companies, and other actors shared the goal of fighting the harms caused by second-hand smoke. Alliance members jointly called for comprehensive EU smoke-free policy and the protection of the political debates from tobacco industry interference. The alliance’s success was enabled by a core group of national and European actors with long-standing experience in tobacco control, who facilitated consensus-building, mobilized allies and synchronized the actions of policy supporters. Representatives of Brussels-based organizations emerged as crucial strategic leaders.

**Conclusions:**

The insights gained and identification of key enablers of successful tobacco control advocacy highlight the strategic importance of investing into tobacco control at European level. Those interested in effective health policy can apply lessons learned from EU smoke-free policy to build effective alliances in tobacco control and other areas of public health.

## Introduction

Broad consensus exists that advocacy and policy engagement form a crucial part of a comprehensive public health strategy and are central to recent achievements in tobacco control.^1^ Scholars and advocates argue that tobacco control alliances have been instrumental in challenging the tobacco industry’s previous political dominance^2–4^ (Weishaar H., Amos A., and Collin J., unpublished data, June 2013) and attaining tobacco control policy successes.^5–9^ In contrast to the largely conceptual and anecdotal literature on advocacy coalitions, there is a dearth of studies which draw on empirical data to systematically investigate coalition-building and identify factors which enhance advocacy coalitions’ impact on policy. Drawing on the development of comprehensive European Union (EU) smoke-free policy as a recent example of successful EU tobacco control policy, this article is the first to empirically analyze a tobacco control alliance in EU policymaking.

The acknowledgement of the critical role of advocacy and coalition-building in achieving effective tobacco control policies is in line with concepts which have been developed to increase understanding of advocacy coalitions, notably Sabatier and Jenkins-Smith’s advocacy coalition framework and Keck and Sikkink’s transnational advocacy network. Sabatier and Jenkins-Smith define advocacy coalitions as groups of political actors who engage in policymaking, share similar values, beliefs and positions and interact regularly in order to influence policy within a given area.^10,11^ Keck and Sikkink’s work focuses on political actors’ efforts to tackle domestic and international policy issues simultaneously by building transnational coalitions.^12^ Political analysts agree that the joint presentation of political interests is useful for demonstrating solidarity and broad support, and attracting decision makers’ attention,^13–16^ thus placing those who build coalitions at a significant advantage when trying to shape policymaking.^12^ The literature, however, is less clear about which characteristics of coalitions and mechanisms of partnership-working enhance advocacy success. Issue, network, and context characteristics have been discussed as contributing factors. Mahoney, for example, argues that a strong urge to combine forces amid a common or immediate threat means that issues of high salience and controversy are particularly likely to generate coalition-building,^16^ while Keck and Sikkink emphasize advocates’ characteristics and relationships as determining the performance and success of coalitions.^12^

This study focuses on the European Council Recommendation on smoke-free environments, a nonbinding document recommending that member states adopt and implement policies to effectively protect their citizens from exposure to second-hand smoke (SHS).^17^ This policy was adopted in November 2009 by the Council of the European Union after almost 3 years of negotiations centered on possible exemptions to the policy and the type of policy for which consensus was achievable. By calling for comprehensive national smoke-free policies, the adopted policy document largely reflects the position of advocates who jointly supported comprehensive EU smoke-free policy (subsequently referred to as the “smoke-free alliance”). By analyzing the composition of the smoke-free alliance, the dynamics of coalition-building, collaboration and leadership, alliance members’ priorities and strategic decisions to pursue unity, and their assessment of the alliance’s success, this paper aims to provide insights into successful public health advocacy and offer lessons for other areas of EU public health policy.

## Methods

A review of policy documents related to the development of the European Council Recommendation on smoke-free environments identified 175 individuals involved in the policy process, their organizational affiliations and policy stance. Stakeholders were assigned to three groups: (a) key actors crucial to the process (e.g., representatives of EU institutions and member states strongly involved in the policy’s development, *n* = 49), (b) stakeholders with considerable interest in the process (e.g., other actors involved in the development of the policy, *n* = 59), and (c) individuals who had shown an interest but had not directly engaged in the process (*n* = 67). Drawing primarily from the first category, interviewees were selected using purposive sampling, aimed at recruiting representatives that varied by type of organization, policy position, geographical remit, national affiliation, and involvement at different stages of the policy process.

Forty-eight individuals were contacted for interview of which six declined and five did not respond. After being contacted via telephone or e-mail and informed about the study’s design, content and purpose, all interviewees provided written informed consent. Thirty-five stakeholders were interviewed across 29 one-to-one and three paired interviews. Interviews were conducted with 18 representatives of health-related organizations (encompassing civil society organizations focusing on tobacco control or public health, scientific institutions centered on tobacco-related research and organizations representing health professionals), five policymakers (politicians and civil servants) and representatives of the tobacco industry (four), social partner organizations (four), the ventilation industry (one) and other commercial sectors (three) (Table 1). Twenty-seven interviews were conducted in person and five via telephone. Interviews included a narrative section on interviewees’ experiences of the policy process, and a semi-structured section exploring their policy positions, collaboration with other actors, coalition leadership and assessment of partnership-working. Interviews were conducted March to July 2011, lasted, on average, 60min and were fully transcribed. A hermeneutic analytical procedure was developed, based upon that described by Bauer,^18^ involving an iterative process of identifying recurring themes, comparison across sub-samples and systematically applying a coding framework to the data set. Interviews were read several times to identify thematic clusters and themes.^19^ Transcripts were then systematically coded according to these themes using QSR NVivo Version 7.^20^ Ethical approval was obtained from the Research Ethics Committee at the School of Health in Social Science of the University of Edinburgh. For a detailed account of the study’s methodology, see Weishaar et al.^21^ Quotes used in the results section indicate the type of organization to which the participant belonged.

**Table 1.** List of Interviewees

Decision makers
Anna Jassem-Staniecka, Policy Officer, European Commission Directorate General for Health and Consumers, Unit 4, Belgium
Alick-James Morris, Policy Officer, European Commission Directorate General for Employment, Social Affairs and Inclusion in the Unit responsible for health, safety and hygiene at work, Luxembourg
Terje Peetso, Policy Officer, European Commission Directorate General for Health and Consumers, Unit 4, Belgium
A representative of the European Economic and Social Committee, Belgium
A representative of an EU institution, Belgium

Representatives of the public health sector
Florence Berteletti-Kemp, Director of the Smokefree Partnership, Belgium
Gregor Breucker, Member of the European Network for Workplace Health Promotion, Germany
Antonella Cardone, Director of the Global Smokefree Partnership, Italy
Magdalena Cedzyńska, Member of International Network of Women Against Tobacco Europe Executive Board, Poland
Norma Cronin, Health Promotion Manager Tobacco Control at the Irish Cancer Society and Member of International Network of Women Against Tobacco Europe Executive Board, Ireland
Sheila Duffy, Chief Executive Action on Smoking and Health Scotland, United Kingdom
Fiona Godfrey, previous Advocacy Director at the European Respiratory Society and European Regional Advisor for Tobacco Control at the International Union against Tuberculosis and Lung Cancer, Belgium
Margaretha Haglund, Tobacco Control Policy Expert at Swedish tobacco control think tank Tobaksfakta and Member of International Network of Women Against Tobacco Europe Executive Board; previously civil servant at the Swedish National Institute for Public Health (Head of National Tobacco Control Programme and Tobacco Control Expert), Sweden
Luk Joossens, Advocacy Officer of the Association of European Cancer Leagues, Belgium
Jean King, Director of Tobacco Control, Cancer Research UK, United Kingdom
Mervi Hara, Director at Finland’s Action on Smoking and Health and Member of International Network of Women Against Tobacco Europe Executive Board, Finland
Martina Pötschke-Langer, Director of the unit for cancer prevention at the German Cancer Research Centre and the WHO Collaborating Centre for Tobacco Control and Member of International Network of Women Against Tobacco Europe Executive Board, Germany
Trudy Prins, Member of International Network of Women Against Tobacco Europe Executive Board; previous Director of the Dutch Expert Centre on Tobacco Control and President of the European Network for Smoking and Tobacco Prevention, Netherlands
Uwe Prümel-Philippsen, Director of the German Federal Association for Prevention and Health Promotion, Germany
Ailsa Rutter, Director of Fresh—Smoke Free North East, UK
Nick Schneider, Science Manager at the unit for cancer prevention at the German Cancer Research Centre and the WHO Collaborating Centre for Tobacco Control, Germany
Gerhard Siemon, Member of the Board of Trustees of the German Lung Foundation, Germany
Friedrich Wiebel, President of the German Medical Action Group Smoking and Health, Germany

Representatives of the tobacco sector
Cynthia Fürste, Corporate Affairs Manager Western Europe British American Tobacco Representation Brussels, Belgium
Bas Tonnaer, Head of Corporate and Regulatory Affairs at British American Tobacco Switzerland SA, Switzerland
Peter van der Mark, General Secretary of the European Smoking Tobacco Association, Belgium
Representative of European Tobacco Wholesalers

Representatives of social partner organizations
Antje Gerstein, Director of the Brussels Representation of the German Employers’ Confederation, Belgium
Helen Hoffmann, Advisor for Social Affairs, European Association of Crafts, Small and Medium-Sized Enterprises, Belgium
Rebekah Smith, Senior Adviser for Social Affairs at BusinessEurope, Belgium
Harald Wiedenhofer, General Secretary of the European Federation of Food, Agriculture and Tourism Trade Unions, Belgium

Representatives of the ventilation sector
Hubert Koch, Director of Dr. Koch Consulting E.K., consultant of the European Alliance For Technical Non-smoker Protection, Germany

Representatives of other commercial sectors
Brussels-based European public affairs expert
Lobbyist
Analyst

## Results

Members of the smoke-free alliance jointly promoted the following three key messages in the policy debates: (a) population health should be improved by protecting EU citizens from the harms caused by SHS; (b) comprehensive smoke-free policies without exemptions are the only way to achieve effective protection from SHS; and (c) given that tobacco companies are the main vector of the tobacco epidemic, tobacco industry representatives should have no opportunity to influence the process of developing smoke-free policies. Table 2 provides selected examples both of how the key messages were communicated and of developments in the policy process which indicate that the messages met with some success. Individual members of the alliance, including representatives of the pharmaceutical sector, health professional bodies and organizations focused on specific diseases, also put considerable efforts into emphasizing smoking cessation as a flanking measure to smoke-free policies. While largely demonstrating agreement on the above three key messages, alliance members were almost evenly divided on preferred type of policy. Half advocated for binding legislation, whereas the other half argued for a nonbinding recommendation (based on the assumption that this policy type would be more likely to receive political support).

**Table 2.** Key Messages, Examples of their Communication and Policy Developments Indicating Success of Such Messages

Key messages	Examples of message communication	Policy developments
Population health should be improved by protecting European Union (EU) citizens from the harms caused by second-hand smoke (SHS).	Submission by the European Network for Smoking Prevention: “The dangerous health effects of secondhand smoke have been documented in over 20 reports…A cautious estimate is that exposure to secondhand smoke kills at least 79 000 people in the EU each year…In addition, secondhand smoke causes a great deal of respiratory diseases and is a major risk factor that exacerbates attacks for people with asthma, allergic illnesses, chronic obstructive pulmonary disease (COPD) and other chronic diseases leading to social and work exclusion and unnecessary illness.”^ 32 ^	The rationale underlying the Council Recommendation on smoke-free environments highlights that SHS is “is a wide spread source of mortality, morbidity and disability in the European Union” and that “people have the right to a high level of health protection and should be protected from exposure to tobacco smoke”.^ 17 ^
	Interview with public health advocate: “We really want to promote health, and health issues.”	
	Interview with tobacco control advocate: “The common interests of saving citizens lives (unites organisations working on tobacco control in Europe). So they are committed to this, and this is what all of them have in common.”	
Comprehensive smoke-free policies without exemptions are the only way to achieve effective protection from SHS.	Submission by ASH England: “Experience from the UK shows that anything less than a comprehensive approach would substantially weaken the smoke-free measure, thus offering less than optimal health protection.”^ 33 ^	The final policy document recommends member states to “provide effective protection from exposure to tobacco smoke in indoor workplaces, indoor public places, public transport and, as appropriate, other public places”^ 17 ^ in line with Framework Convention on Tobacco Control (FCTC) article 8. The guidelines to FCTC article 8 state that effective measures “require the total elimination of smoking and tobacco smoke in a particular space or environment in order to create a 100% smoke-free environment.”^ 35 ^
	Submission by the German Cancer Research Centre: “Article 8 of the WHO Framework Convention (protection from exposure to tobacco smoke) obligates Parties to take effective steps to provide protection from exposure to tobacco smoke. Effective measures require the total elimination of smoking and tobacco smoke in a particular space or environment in order to create a 100% smoke-free environment. There is no safe level of exposure to tobacco smoke…Approaches other than 100% smoke-free environments, including ventilation, air filtration and the use of designated smoking areas…have repeatedly been shown to be ineffective.”^ 34 ^	
	Interview with tobacco control advocate: “So the framing of the problem, we were all in agreement about…We wanted 100% smoke-free with no exemptions. And we were…very, very clear on that.”	
Given that tobacco companies are the main vector of the tobacco epidemic, tobacco industry representatives should have no opportunity to influence the process of developing smoke- free policies.	Submission by the European Public Health Alliance: “We would like to reiterate that the tobacco industry must be excluded from smoke free policy debates because of the unique role of its products in causing harm and because of its track record of deceptive behaviour.”^ 36 ^	In the consultation meeting with stakeholders initiated by the European Commission, public health and civil society representatives objected to being consulted in a joint meeting with tobacco industry representatives, arguing that tobacco manufacturers and their allies should not be treated as legitimate stakeholders. Accordingly, the meeting minutes indicate that two separate meetings took place, one with tobacco and ventilation industry representatives, and the other with health experts, civil society and social partners.
	Interview with tobacco control advocate: “Tobacco companies can put in their views in a paper exercise but that’s as far as it should go. They should not be treated like normal stakeholders and that’s made very clear in the FCTC article 5.3.”	
	Interview with analyst: “The argumentation from the NGOs and researchers was that the tobacco industry in earlier discussions had, in their opinion, ruined the complete discussion by coming up with all sorts of nonsensical arguments.”	

The data clearly indicate that members of the smoke-free alliance recognized a need to demonstrate unity on the issue of EU smoke-free policy and were committed to collaboration to enhance advocacy success. Interviewees perceived coalition-building to be crucial for achieving policy success and highlighted the value of collaboration in influencing policymakers. This was described by one interviewee as reducing “complexity,” enhancing “power of persuasion” and demonstrating the presence of “strong interests”:

*Politicians always like these kinds of alliances because they say: ‘Preliminary debates have been held. Positions have been clarified. And…they have relieved us of some of the work.’ Because politicians always have to balance the various stakeholders’ interests. And the better they have aligned their interests, the easier it is.* (representative of the ventilation sector)

Table 3 outlines the four key factors identified as contributing to the alliance’s positive impact on policy: (a) composition, (b) priorities and pursuit of unity, (c) collaboration, and (d) leadership and coordination.

**Table 3.** Factors Identified as Contributing to the Alliance’s Impact on Policy

Factors identified as contributing to the alliance’s impact on policy	Specification
1. Composition of the alliance	Different types of organization
	Issue-specific organizations and organizations with a broader remit
	Actors with resources to financially support the campaign
	Input from researchers and health professionals
2. Priorities and pursuit of unity	Shared vision about reducing harms from tobacco and second-hand smoke
	Consensus on favored policy measures
	Agreement on key lobbying messages
	Shared identification of, and resistance to, opposition
3. Collaboration	Personal interaction, long-term collaboration and trust
	Information exchange
	Pooling of resources
4. Leadership and coordination	Lead actor(s) with understanding of the policy issue and policy process and ability to provide strategic direction
	Core group of actors committed to the issue and able to engage in advocacy and levy resources
	Ability to mobilize support
	Good working relationships between organizations supporting the issue across different levels of governance (local, national, European Union EU, global)

### Composition of the Alliance

The smoke-free alliance was comprised of advocacy organizations with an interest in public health, scientific institutions, professional organizations and pharmaceutical sector organizations. It included organizations centered on national, European and global tobacco control (e.g., national nonsmokers’ initiatives, the Framework Convention Alliance, the International Network of Women Against Tobacco) and broader health-related issues (e.g., the European Public Health Alliance, the British Medical Association, pharmaceutical companies). Tobacco control advocates indicated that they prioritized recruiting diverse allies to convey broad public support for comprehensive EU smoke-free policy; the alliance’s heterogeneity reflected a strategic effort to “develop a coalition which was not only tobacco control” (tobacco control advocate) and to present a “united front” across public health (public health advocate). Such efforts seemingly met with success, illustrated by tobacco industry representatives and politicians referring to the supporters of comprehensive EU smoke-free policy as “a one-issue movement” representing “the voice of the health sector.”

Key strategic decisions included coalition-building with the pharmaceutical sector. While civil society organizations’ messages emphasized the need to adopt smoke-free policies, pharmaceutical industry messages focused on smoking cessation as a flanking measure to such policies. However, the pharmaceutical sector’s input was valued as increasing the alliance’s financial resources, opening up additional opportunities for communication with decision makers and enhancing the alliance’s visibility. For example, dinner debates were held in the European Parliament, funded by the pharmaceutical sector and co-organized with European health organizations and professional bodies.^22,23^ Interviewees viewed such events as helpful in raising awareness, facilitating debates about comprehensive smoke-free policy, and increasing the prominence of the smoke-free alliance.

Organizations representing researchers, academics and professionals also showed considerable engagement and close collaboration with advocacy organizations. These actors were seen as enhancing the credibility of the smoke-free alliance, being perceived within the coalition as conveying “authority” and being “taken…more seriously than an advocacy group.” A representative of a scientific institution stressed that decision makers particularly valued the input and expertise provided by academics and professionals, assigning more weight to their positions and statements.

### Priorities and Pursuit of Unity

Alliance members shared a common motivation to fight the harms caused by SHS, with members describing unity based on a “shared vision” to “save citizens’ lives” (public health advocates) and “change society for the better” (European public affairs expert). This “common interest” (tobacco control advocate) resulted in two lobbying messages being consistently advanced by alliance members: the need for comprehensive smoke-free policy without exemptions and protection of the policy process from tobacco industry interference. Interviewees described earlier international negotiations for the World Health Organization Framework Convention on Tobacco Control (FCTC) as easing development of a clear set of agreed measures:

*[Reaching agreement] is not usually difficult. Especially now with the FCTC, because that is a very strong framework for us…The FCTC is a good blueprint.* (public health advocate)

International consensus about effective tobacco control strategies helped advocates to publicly advance “a unified position on SHS” (tobacco control advocate) in the EU context.

Alliance members were also united in their identification of the tobacco industry as an opponent and the view that the industry’s engagement in the debates would threaten the adoption of effective policy. Tobacco control advocates, in particular, perceived the tobacco industry and its representatives as “a clear and tangible enemy”, requiring a strong united front and response. Referring to the long history of tobacco industry efforts to undermine tobacco control policies, one advocate reflected that “maybe the fact that the tobacco companies (had previously) behaved so outrageously helped to unite us.” Advocates also identified an imbalance of resources between “the brilliantly positioned tobacco industry” and those working in tobacco control; the risk that tobacco companies would exploit any dissent created an imperative to collaborate:

*We need to be united in what we are prioritising at any one time because we have very limited resources. Also, if there are different voices or different policies being promoted, the opposition will do all they can to exploit that. So it’s very important that we can reach an agreed position and agreed policies.* (tobacco control advocate)

### Collaboration

Trust emerged as a crucial element of collaboration among members of the smoke-free alliance. Advocates reported building on their long-standing experience of jointly lobbying for national, European and international public health policies, which had promoted consensus and trust, refined advocacy strategies and created strong personal and professional relationships:

*We were a group of people who, by that point, had been working together for a long time on tobacco control and other health issues sometimes. We knew each other, we trusted each other, we respected each other’s judgment.* (public health advocate)

Formal organizational networks, online communities, and international conferences were seen as crucial opportunities to reinforce existing contacts and as important venues for information exchange and policy discussion. Alliance members described using their networks to gather opinions about how to react to the policy proposal, obtain feedback and advice, mobilize allies to submit responses and exchange draft texts. Health advocates agreed that “sharing messages, information and intelligence” had been “vital” in achieving success and mentioned several practical benefits of coalition-building, including knowledge transfer, reduced workload and increased efficiency:

*The biggest advantage was the fact that we had access to each other. So we could tap into each other’s resources and each other’s knowledge…If you put all the intelligence of the people together it’s more than just the sum of all the people who are there. It adds something.* (public health advocate)

### Leadership and Coordination

A clear sign of key advocates’ recognition of the strategic value of pooling resources was the establishment of the European Smokefree Partnership (SFP). Created in 2005 by European and national tobacco control organizations in response to emerging discussions on EU smoke-free policy, this Brussels-based organization, aimed at promoting tobacco control advocacy at the EU level, emerged as a key player in the debates. The SFP was highlighted by several interviewees as important in disseminating information, driving the agenda, mobilizing actors, building partnerships and providing strategic direction. Viewed as the focal point of the alliance, the SFP was part of a group of representatives of European and national organizations who held key roles at national or European levels and were experienced in working together in EU lobbying. These individuals were described by several public health advocates as forming an informal European “strategy group”, which had held regular teleconference discussions of EU policy developments and strategies since the 1990s. The strategy group members’ knowledge of and commitment to tobacco control and their relationships with each other were regarded as crucial assets. They subsequently led the lobbying campaign at national and European levels, mobilizing other organizations, facilitating collaboration between supporters and coordinating action, thus allowing the sharing of resources and increasing efficiency.

Interviewees highlighted the importance of tobacco control advocacy being led by Brussels-based organizations, whose proximity allowed them to keep “their finger on the pulse” (public health advocate) and “be part of the day-to-day business with regard to information and the monitoring process” (tobacco industry representative). Also perceived as important were the lead organizations’ and particularly the SFP’s thorough understanding of the EU policy process, ability to assess likely support and opposition, and tactical approach to advocacy. Leadership and guidance from Brussels-based organizations was seen as crucial in helping member state advocates tailor messages to national decision makers and lobby for comprehensive EU smoke-free policy:

*Someone has to be up there doing the guidance…to the countries: ‘Now it’s the right time to do this, to write to your ministry. Now it’s the right time to talk to your MEPs in Brussels.’ Someone up there on the Brussels level has to give these instructions to the countries.* (national tobacco control advocate)

## Discussion

This article analyzes coalition-building and collaboration among supporters of effective EU tobacco control policy in the run-up to the Council Recommendation on smoke-free environments. It complements previous work on advocacy coalitions which highlights the advantages of coalitions in terms of accessing, sharing and disseminating information, pooling resources, enhancing advocates’ ability to demonstrate solidarity, agreement and support for a policy position, garnering decision makers’ support and influencing policy processes.^12,16^ Our analysis provides evidence of the dynamics of the diverse alliance which supported comprehensive EU smoke-free policy and included advocacy organizations, scientific institutions, professional organizations, and pharmaceutical sector organizations. At the core of the alliance, leading tobacco control advocates drove strategic decisions regarding coalition-building and the mobilization of actors with a broader remit. This resulted in the alliance being perceived as broadly representing the health sector, rather than a narrow tobacco control interest.

While heterogeneous, the alliance shared the common goal of fighting harms caused by SHS and agreed on and jointly advanced a small number of clear key messages: the need to protect EU citizens from SHS by implementing comprehensive EU smoke-free policy without exemptions and to safeguard the policy process from tobacco industry interference. While, in line with the advocacy coalition framework,^10^ normative beliefs about the primacy of health can be seen as the “glue” that held alliance members together, strategic agreement on key messages and actions and demonstrating unity seemed to be similarly important. Policy debates were thus focused on key broad messages, with disagreement on more specific issues (e.g., on stakeholders’ favored policy type or on flanking measures) subordinated given the priority afforded to demonstrating unanimity. The analysis highlights that previous collaboration on tobacco control initiatives helped coalition members to agree on lobbying messages and contributed to successful coalition-building and collaboration on EU smoke-free policy. That advocates knew and relied on each other and had experience of working together was seen as facilitating alliance formation and cooperation, mirroring research by Klijn and colleagues about the importance of trust in governance networks.^24^ Awareness of limited resources, previous experience of tobacco industry successes in preventing effective public health policy and the common perception of the tobacco industry as a powerful enemy, served to unify and enhance cohesion among health advocates.

The analysis highlights the role of national and European lead organizations in facilitating effective advocacy and coalition-building for public health. While organizations such as the Bureau for Action on Smoking Prevention and the European Network for Smoking Prevention have been recognized as leaders of advocacy campaigns to advance previous EU tobacco control legislation,^25^ our study shows that in this recent case, SFP was crucial in coordinating the actions of organizations dispersed across member states from its base in Brussels. National lead organizations, on the other hand, focused on mobilizing allies in their respective member states. Such findings not only echo accounts of the importance of lead organizations within successful tobacco control alliances at national level,^6,26,27^ but highlight the importance of European advocacy leadership. The analysis also shows that in addition to relying on SFP’s synchronization, alliance leadership was shared among a small number of organizations that devoted a considerable proportion of their resources to advancing a tobacco control agenda and coordinating other actors. The close interaction, consensus-building and collaboration among this core group of national and Brussels-based advocates appeared as a key strength of the alliance, contributing to its ability to disseminate information and enlist allies, increasing its reach across national and European levels.

This study identifies common values, unity among advocates and agreement on strategies as crucial factors for successful coalition-building and public health advocacy. The specific features and challenges of advocacy alliances identified in this study also highlight the transnational dimension of alliances that operate in the EU context. This suggests that concepts like Keck and Sikkink’s transnational advocacy network^12^ might prove valuable when analyzing public health governance, transnational alliances, and the interactions that are characteristic of the EU policy process. Our analysis further highlights that organizations experienced in advocating and negotiating EU policymaking are particularly important to public health alliances operating at EU level. In line with the image of Brussels as an “insider’s town”^28^ or a “bubble,”^29^ the findings indicate that the complexity and intricacies of the EU policy process increase the significance of Brussels-based actors who can “translate”, mediate and provide strategic guidance on “Brussels politics”. By identifying the considerable demands which such organizations are confronted with, including the need to keep a multitude of organizations informed and coordinate differing interests, the analysis suggests that leaders of European alliances face particular challenges. Concepts of political entrepreneurship, which focus on the key characteristics of actors with an ability to direct political processes,^30,31^ might usefully inform further analyses of the role of lead organizations in EU public health alliances.

This study identifies a number of features which appear to advance successful public health advocacy: mobilizing diverse actors beyond those with a circumscribed interest in a specific policy; agreeing core values and key messages; collaborating by exchanging information and pooling resources; and coordination by designated campaign leaders. By identifying factors contributing to successful public health advocacy, the article provides valuable lessons for those with an interest in effective public health policy and has potential to inform the development of effective public health advocacy at European and national levels. The analysis of advocacy on EU smoke-free policy might help advocates engaged in on-going debates on tobacco control and other policy areas, including alcohol and food policy, to build successful alliances for public health.

## Funding

This work was supported by the UK Centre for Tobacco and Alcohol Studies (UKCTAS) , a UK Clinical Research Collaboration Public Health Research Centre of Excellence. Funding under the auspices of the UK Clinical Research Collaboration from British Heart Foundation, Cancer Research, UK, Economic and Social Research Council, Medical Research Council, and Department of Health is gratefully acknowledged. The views expressed in this paper are those of the authors and not necessarily the funders. JC receives support from the National Cancer Institute of the US National Institutes of Health (grant no R01 CA160695-01).

## Declaration of Interests

*HW is co-chair of the Scottish Tobacco Control Alliance’s Research Group. JC is a member of the Tobacco Advisory Group for Cancer Research UK. AA is member of the board of the International Network of Women Against Tobacco Europe*.
